# Caveolin‐1 suppresses hippocampal neuron apoptosis via the regulation of HIF1α in hypoxia in naked mole‐rats

**DOI:** 10.1002/cbin.11890

**Published:** 2022-09-02

**Authors:** Wenjing Yang, Wenqing Wu, Ying Zhao, Yu Li, Chengcai Zhang, Jingyuan Zhang, Chao Chen, Shufang Cui

**Affiliations:** ^1^ Department of Laboratory Animal Sciences, School of Basic Medicine Naval Medical University Shanghai China; ^2^ Department of Laboratory Animal Center Academy of Military Medical Sciences Beijing China; ^3^ Shanghai Laboratory Animal Research Center Shanghai China

**Keywords:** brain, caveolin‐1, hippocampal neuron, hypoxia, hypoxia inducible factor‐1α, naked mole‐rats

## Abstract

Naked mole‐rats (NMRs) (*Heterocephalus glaber*) are highly social and subterranean rodents with large communal colonies in burrows containing low oxygen levels. The inhibition of severe hypoxic conditions is of particular interest to this study. To understand the mechanisms that facilitate neuronal preservation during hypoxia, we investigated the proteins regulating hypoxia tolerance in NMR hippocampal neurons. Caveolin‐1 (Cav‐1), a transmembrane scaffolding protein, confers prosurvival signalling in the central nervous system. The present study aimed to investigate the role of Cav‐1 in hypoxia‐induced neuronal injury. Western blotting analysis and immunocytochemistry showed that Cav‐1 expression was significantly upregulated in NMR hippocampal neurons under 8% O_2_ conditions for 8 h. Cav‐1 alleviates apoptotic neuronal death from hypoxia. Downregulation of Cav‐1 by lentiviral vectors suggested damage to NMR hippocampal neurons under hypoxic conditions in vitro and in vivo. Overexpression of Cav‐1 by LV‐Cav‐1 enhanced hypoxic tolerance of NMR hippocampal neurons in vitro and in vivo. Mechanistically, the levels of hypoxia inducible factor‐1α (HIF‐1α) are also increased under hypoxic conditions. After inhibiting the binding of HIF‐1α to hypoxia response elements in the DNA by echinomycin, Cav‐1 levels were downregulated significantly. Furthermore, chromatin immunoprecipitation assays showed the direct role of HIF1α in regulating the expression levels of Cav‐1 in NMR hippocampal neurons under hypoxic conditions. These findings suggest that Cav‐1 plays a critical role in modulating the apoptosis of NMR hippocampal neurons and warrant further studies targeting Cav‐1 to treat hypoxia‐associated brain diseases.

## 1 INTRODUCTION

Hypoxia ischaemic encephalopathy, such as hypoxic stroke, has become one of the major life‐threatening conditions in the world. There are more than 5 million people die from stroke and more than 5 million disabilities resulted from stroke per year (Norrving & Kissela, 2013; Toman et al., 2019). When most vertebrates suffered from ischemia, even minutes of lacking of oxygen and nutrients leads to irreversible apoptosis and necrosis of a large number of neurons, as well as paralysis and other dysfunction (Buck & Pamenter, 2018; Sun et al., 2020). Therefore, efficient prevention measures aim to prevent neuronal apoptosis and necrosis, will be a great boon for patients suffering from hypoxic stroke (Baker et al., 1998; Bano & Nicotera, 2007; Clark et al., 1994; Lipton, 1999).

Certain vertebrates tolerate both transient and chronic hypoxic environments, such as freshwater turtles, seals and whales, and African naked mole‐rats (NMRs) (Burns et al., 2007; Christian et al., 2008; Jackson & Ultsch, 2010; Larson et al., 2014). There were many outstanding characteristics in NMRs to adapt the environment, to protect against cancer neurodegenerative diseases, chemical pain and hypoxia (Edrey et al., 2013; Liang et al., 2010; Schuhmacher et al., 2018; Zhao et al., 2018). Naked mole rats live in poorly ventilated underground burrows with higher concentrations of carbon dioxide and lower concentrations of oxygen (Holtze et al., 2018). Adaptations to this environment makes them extremely resistant to hypoxia, even in the laboratory, surviving for weeks in 8% O_2_, and even up to 18 min in anoxia conditions (Chung et al., 2016; Holtze, Braude, et al. 2018, Ilacqua et al., 2017; Kim et al., 2011; Park et al., 2017). These findings suggest that naked mole rats have evolved physiological mechanisms to adapt to a constant low oxygen and high carbonic acid environment (Holtze et al., 2018). Unique circulatory and metabolic functions may help them cope with the environments lacking oxygen (Holtze et al., 2018), such as reducing the basic metabolic rate (Holtze et al., 2018), such as reducing the basic metabolic rate (McNab, 1979), increasing the ability of haemoglobin to bind oxygen, and enhancing acid buffering capacity (Johansen et al., 1976). Compared with other tissues, the brain is extremely sensitive to hypoxia due to its high metabolic rate and lack of nonoxidative energy stores (Siesjö, 1988). On the other hand, NMR neurons evolved to find new solutions to survive in hypoxic conditions. In vitro experiments also show that hippocampal slices from NMRs can tolerate acute hypoxic conditions (Larson & Park, 2009). Unfortunately, to date, the mechanisms for the hypoxic adaptation of NMR hippocampal neurons have not been illustrated completely. Once the mechanisms that protect the NMR brain from hypoxia are found, this may provide critical insights for human diseases involving hypoxia, such as ischaemic stroke.

Hypoxic adaptation of mammalian cells mainly depend on the function of the transcription factor heterodimer hypoxia‐inducible factor (HIF) family, which consists of α and β subunits (Kaelin, 2002; Semenza, 2003). The catalytic HIF1α subunit is oxygen‐sensitive, which targets ubiquitin mediated destruction via von Hippel‐Lindau (VHL) tumour suppressor‐containing E3 ubiquitin ligase (Kaelin, 2002). Interestingly, VHL binding regions in NMR origined HIF1α of showed a unique exchange of T407I and V166I, which enables HIF1α to escape from degradation under normoxic conditions (Kim et al., 2011). Currently, whether HIF‐1 α is involved in regulating NMR brain hypoxia adaptation still remains undiscovered.

Caveolae, primarily identified as small flask‐shaped invaginations of the plasma membrane, ranging from 50 to 100 nm (Zhang et al., 2016), with a molecular weight of 21–24 kDa, is responsible for caveolae formation, which participates in intracellular biological events such as regulating signal transduction and cell differentiation, proliferation and apoptosis (Parton & del Pozo, 2013; Parton & Simons, 2007) by binding with some proteins related to signalling via scaffolding domains (CSDs) (Abulrob et al., 2004). Cav‐1 knocking off in animal models results in loss of the organelle (Drab et al., 2001). However, whether Cav‐1 is expressed in the brains of NMRs and participates in hypoxia tolerance remains unknown.

In the present study, we found that Cav‐1 is expressed in hippocampal neurons. Cav‐1 expression is increased after hypoxia treatment. We further investigated the mechanisms by which Cav‐1 regulates the hypoxia tolerance of NMR hippocampal neurons. In addition, HIF‐1α modulates Cav‐1 expression in NMR hippocampal neurons after hypoxia. Collectively, these results suggest that especially high levels of Cav‐1 in NMR hippocampal neurons during hypoxia may be neuroprotective.

## 2 MATERIALS AND METHODS

### 2.1 Animals

Adult NMRs, C57BL/6J mice, new‐born NMRs and mice were provided by the Laboratory Animal Department of Naval Medical University (Shanghai, China). All animals were cared for and used in accordance with the Chinese laws for animal experimentation and regulations. All animal procedures were approved by the Naval Medical University Animal Care and Use Committee. NMRs were bred in‐house and maintained in mouse/rat cages connected by tunnels of different lengths as previously reported (Yang et al., 2017). The room was warmed to 30°C and humidified, and red lighting (08:00–16:00) was used. Mice were maintained on a normal 12‐h light/dark cycle with food and water available ad libitum. For acute hypoxic stress, animals were placed in an Animal Hypoxia Chamber (Tawang Intelligent Technology). Adult NMRs (age: 15–18 months) and C57BL/6J mice (8–10 weeks) were treated with acute hypoxia (8% O_2_) for 8 h. Meanwhile, control groups were subjected to normoxic conditions (21% O_2_) for 8 h.

### 2.2 Cell culture

Primary neurons isolated from the hippocampus of postnatal 24 h NMRs were cultured in Dulbecco modified Eagle medium (DMEM) containing 10% heat‐inactivated foetal bovine serum (Life Technologies), 2 mM glucose, 0.0025% Glutamax, and 0.2 mg/ml primocin (Thermo Fisher Scientific). Primary neurons isolated from the hippocampus of postnatal 24 h mice were cultured in DMEM containing 10% heat‐inactivated foetal bovine serum (Life Technologies), 4.5 g/L glucose, 0.0025% Glutamax, and 0.2 mg/ml primocin (Thermo Fisher Scientific). Four hours later, the medium was replaced with neurobasal A medium supplemented with B27 (2%), 250 mM Glutamax, and penicillin/streptomycin (1%). NMR neurons were maintained at 33°C in 21% O2/5% CO_2_. Half of the medium was renewed every 48–72 h before further experiments. For hypoxic stress, hippocampal neurons from NMRs were maintained at 33°C (5% CO_2_, 8% O_2_), and hippocampal neurons from NMRs were maintained at 37°C (5% CO_2_, 8% O_2_), for the indicated periods of time in the absence or presence of 20 mM echinomycin (Sigma–Aldrich).

### 2.3 Immunofluorescence assay

The immunofluorescence assay was performed as previously reported (Yang et al., 2015). Briefly, after fixation in 4% paraformaldehyde/phosphate‐buffered saline (PBS) for 10 min at room temperature (RT) and permeabilized with 0.3% Triton X‐100/PBS for 5 min. After blocking with 1% bovine serum albumin (BSA)/PBS for 20 min, the cells were incubated with primary antibodies, including Cav‐1 (1:100; Proteintech), HIF‐1α (1:100; Proteintech), NeuN (1:100; Proteintech), and Caspase3 (1:100; Proteintech), at 4°C overnight. After three washes, the cells were incubated with anti‐mouse or anti‐rabbit fluorescent secondary antibodies (1:200; Jackson Lab) at RT for 1 h. The nuclei were stained with Hoechst 33342 (1:1000; Sigma) for 20 min. Photos were obtained using a Leica microscope (Germany).

Brain slices (4–6 μm) from NMR and mouse brains were permeabilized with 0.1% Triton X‐100 for 10 min and blocked with 1% BSA/PBS for 20 min at RT. For immunofluorescence labelling studies, a variety of primary antibodies were applied, including Cav‐1 (1:100; Proteintech), NeuN (1:100; Proteintech), Caspase3 (1:100; Proteintech), and HIF‐1α (1:100; Proteintech), and fluorescent secondary antibodies (1:200; Jackson Lab) at RT for 1 h. Photos were obtained at RT using a Leica microscope (Germany).

### 2.4 Western blotting analysis

Western blotting analysis was performed as described previously (Yang et al., 2015). Proteins were extracted from cultured cells or hippocampal tissues that were lysed in ice‐cold RIPA buffer (Beyotime) containing protease inhibitors (Roche). Homogenates were centrifuged at 12,000 rpm for 30 min at 4°C, and supernatants were collected. After quantification by NanoDrop One before electrophoresis. Samples were separated by sodium dodecyl sulphate polyacrylamide gel electrophoresis using 12% acrylamide gels and transferred to polyvinylidene difluoride membranes (Millipore) by electroelution. Membranes were blocked in 10% skimmed milk and incubated with primary antibodies overnight at 4°C. Proteins were detected by the following antibodies: anti‐Cav‐1 (1:1000; Protein Tech), anti‐HIF‐1α (1:1000; CST), anti‐caspase3 (1:1000; Protein Tech), and horseradish peroxidase conjugated anti‐glyceraldehyde 3‐phosphate dehydrogenase (1:10,000; Protein Tech). Primary antibodies were visualised using secondary antibodies conjugated to horseradish peroxidase (1:10,000; Protein Tech) and enhanced chemiluminescence reagent (Tanon, China). The chemiluminescent signals were captured by the Gel Doc gel imaging system (Tanon, China). All experiments were performed independently at least three times. The blots were scanned with an Odyssey imager (LI‐COR Biosciences), and band intensity was determined with a Quantity One System (Bio–Rad).

### 2.5 Lentivirus

NMR Cav‐1 cDNA (XM_004865265.3) was amplified and subcloned into the BamH I/Age I sites of the pHBLV‐U6‐MCS‐CMV‐ZsGreen‐PGK‐PURO vector (Hanheng Biology). For the generation of short hairpin RNA (shRNA) lentivirus, complementary oligonucleotides were annealed and inserted into the BamHI/EcoR I sites of the pHBLV‐U6‐MCS‐CMV‐ZsGreen‐PGK‐PURO vector. The complementary oligonucleotides of pHBLV‐Cav‐1 shRNA were 5′‐GATCCGTCACCACCTTCACTGTGACGAAATATTCAAGAGATATTTCGTCACAGTGAAGGTGGTGATTTTTTG‐3′ and 5′‐AATTGAAAAAATTCTCCGAACGTGTCACGTAATCTCTTGAATTACGTGACACGTTCGGAGAACG‐3′ (Target Seq TCACCACCTTCACTGTGACGAAATA). The complementary oligonucleotides of pHBLV‐scrambled shRNA were 5′‐GATCCGTTCTCCGAACGTGTCACGTAATTCAAGAGATTACGTGACACGTTCGGAGAATTTTTTC‐3′ and 5′‐AATTGAAAAAATTCTCCGAACGTGTCACGTAATCTCTTGAATTACGTGACACGTTCGGAGAACG‐3′ (Target Seq TTCTCCGAACGTGTCACGTAA). Lentivirus particles were used to infect neurons as previously described 48 h before further investigation (Larson & Park, 2009). The downregulation effects of NC and 3 kinds of shRNA against NMR Cav1 were analysed by western blotting analysis (Figure S1a and SIb).

Mouse Cav‐1 cDNA (NM_007616.4) was amplified and subcloned into the BamH I/Age I sites of the pHBLV‐U6‐MCS‐CMV‐ZsGreen‐PGK‐PURO vector (Hanheng Biology). For the generation of shRNA lentivirus, complementary oligonucleotides were annealed and inserted into the BamHI/EcoR I sites of the pHBLV‐U6‐MCS‐CMV‐ZsGreen‐PGK‐PURO vector. The pHBLV‐Cav‐1 shRNA targets the sequence of CGAAGGACTAACTCTAAGT (Lee et al., 2015).

### 2.6 Animal model

LV‐EGFP‐Cav‐1, LV‐EGFP (i.e., Control), LV‐sh‐Cav‐1, and LV‐noncoding RNA (LV‐NC) viruses were produced by Hanheng Bio Companies. Viruses were injected into the hippocampus of both cerebral hemispheres by brain stereotactic injection instruments. For mice, the injection position was based on the following stereotactic coordinates (mm from bregma): dentate gyrus (AP: −2 mm; L: −1.5 mm; DV: +1.75 mm) of both cerebral hemispheres according to the stereotaxic atlas of Franklin and Paxinos (KB and G, 2001). For the adult NMRs (18 months), virus was injected into the hippocampus dentate gyrus (AP: −2 mm; L: −1.4 mm; DV: +1.75 mm). One microlitre of 4–8 × 10^9^ viral particles was injected slowly (0.1 μl, 30 s) into each side (Xiao et al., 2006). Animals in the sham control groups were injected with diluent such as volume of experiment groups in the same place. The areas of hippocampus were taken for further investigations, and CA1 regions were shown in the Immunofluorescence staining.

### 2.7 Flow cytometry

Cell death was evaluated using Annexin V‐APC/7‐AAD double staining or an Annexin V‐FITC/PI Apoptosis Detection Kit according to the manufacturer's protocol (Biolegend). Briefly, 1.0 × 10^6^ cells/ml were washed in ice‐cold PBS, centrifuged at 1000 rpm for 5 min, resuspended in APC binding buffer and incubated with AnV conjugated to APC and with 7‐AAD or PI. After 15 min of incubation in the dark, cells were diluted in 300 μl of binding buffer and analysed by flow cytometry (Becton Dickinson). For each test, 1.0 × 10^6^ cells were used, and data on at least 10,000 events were obtained using Cell Quest software (Becton Dickinson).

### 2.8 Chromatin immunoprecipitation (ChIP) assay

ChIP assays were performed using a ChIP assay kit (Upstate Biotechnology). Cells incubated under hypoxic conditions for 8 h were fixed with 1% formaldehyde/PBS and sonicated to obtain 500–1000‐bp DNA fragments. Chromatin was immunoprecipitated with 5 mg of anti‐HIF‐1a (Novus, NB100‐134) or rabbit immunoglobulin G. The immunoprecipitated DNA was amplified with a primer pair specific for the Cav‐1 promoter (F, 5′‐TGCTCTCGGTTATGTTTCC‐3′ and R, 5′‐CAGTCCGTGCTTGTCTCCT‐3′).

### 2.9 Statistical analysis

The experimental data were analysed using Student's *t* test, or one‐way or two‐way analysis of variance followed by Bonferroni‐*t* test with SPSS 26 statistical software. All experiments were repeated at least three times independently. All *p* values less than .05 were considered significant.

## 3 RESULTS

### 3.1 Cav‐1 levels are upregulated in hippocampal neurons under hypoxia in a time‐dependent manner in vitro

**Figure 1:**
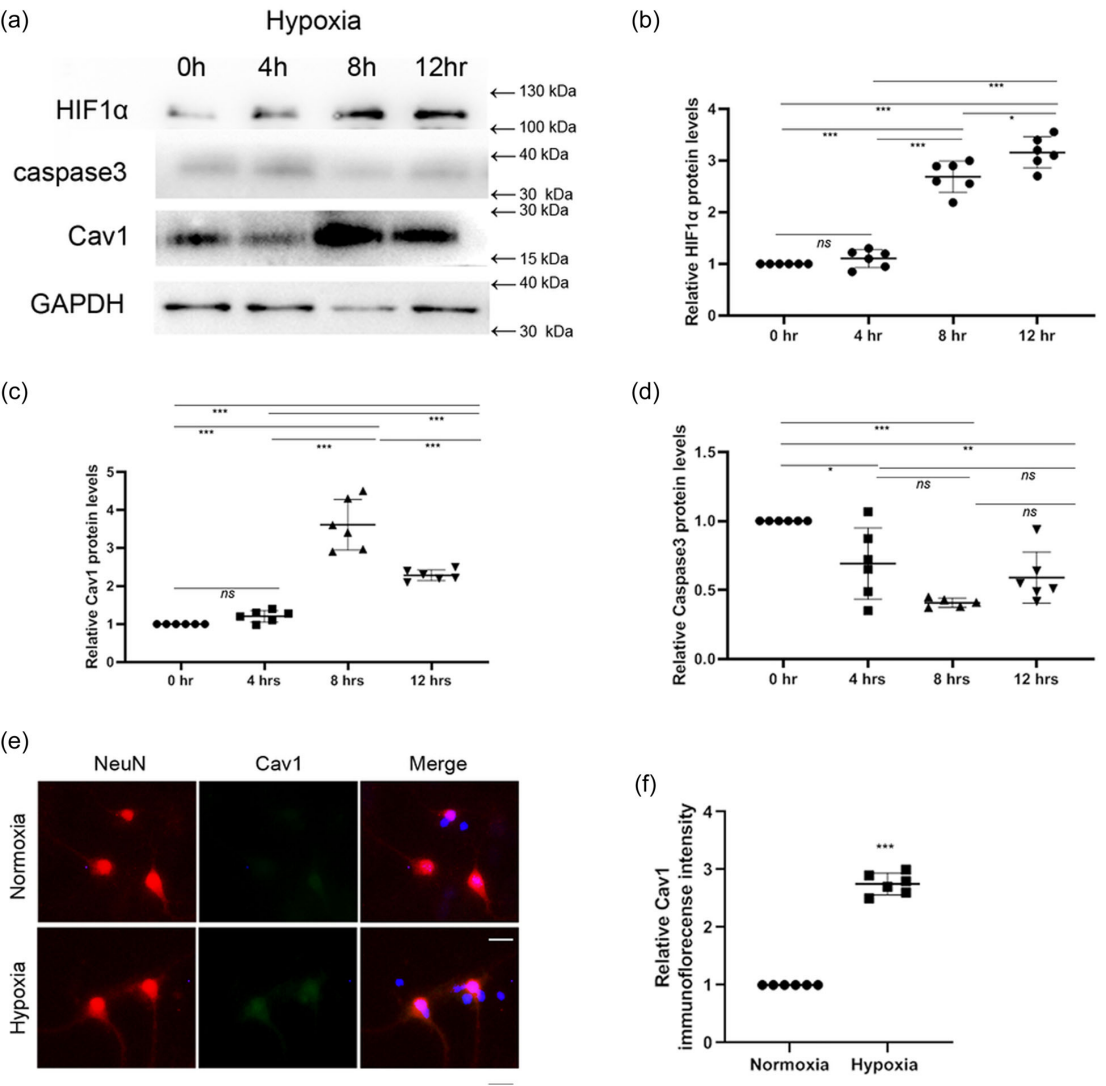
Cav‐1 pattern of NMR hippocampal neurons during hypoxia and normoxia (a) Primary hippocampal neurons were incubated under hypoxia for 0, 2, 4, 8 and 12 h. Cav‐1, caspase3, HIF‐1α, and GAPDH protein levels were investigated by western blotting analysis. (b–d) The results are shown as the mean ± *SEM* (*n* = 6 per group). (e) Primary NMR hippocampal neurons were cultured under hypoxia or normoxia for 8 h, and the level of cellular Cav‐1 was examined by immunocytochemistry (scale bar: 20 μm). (f) statistics for relative Cav1 immunoflorecense intensity in (Figure 1e). The results are shown as the mean ± *SEM* (*n* = 6 per group). **p < .01, *** *p* < .001. GAPDH, glyceraldehyde 3‐phosphate dehydrogenase; HIF, hypoxia inducible factor‐1α; NMR, naked mole‐rats

**Figure 2:**
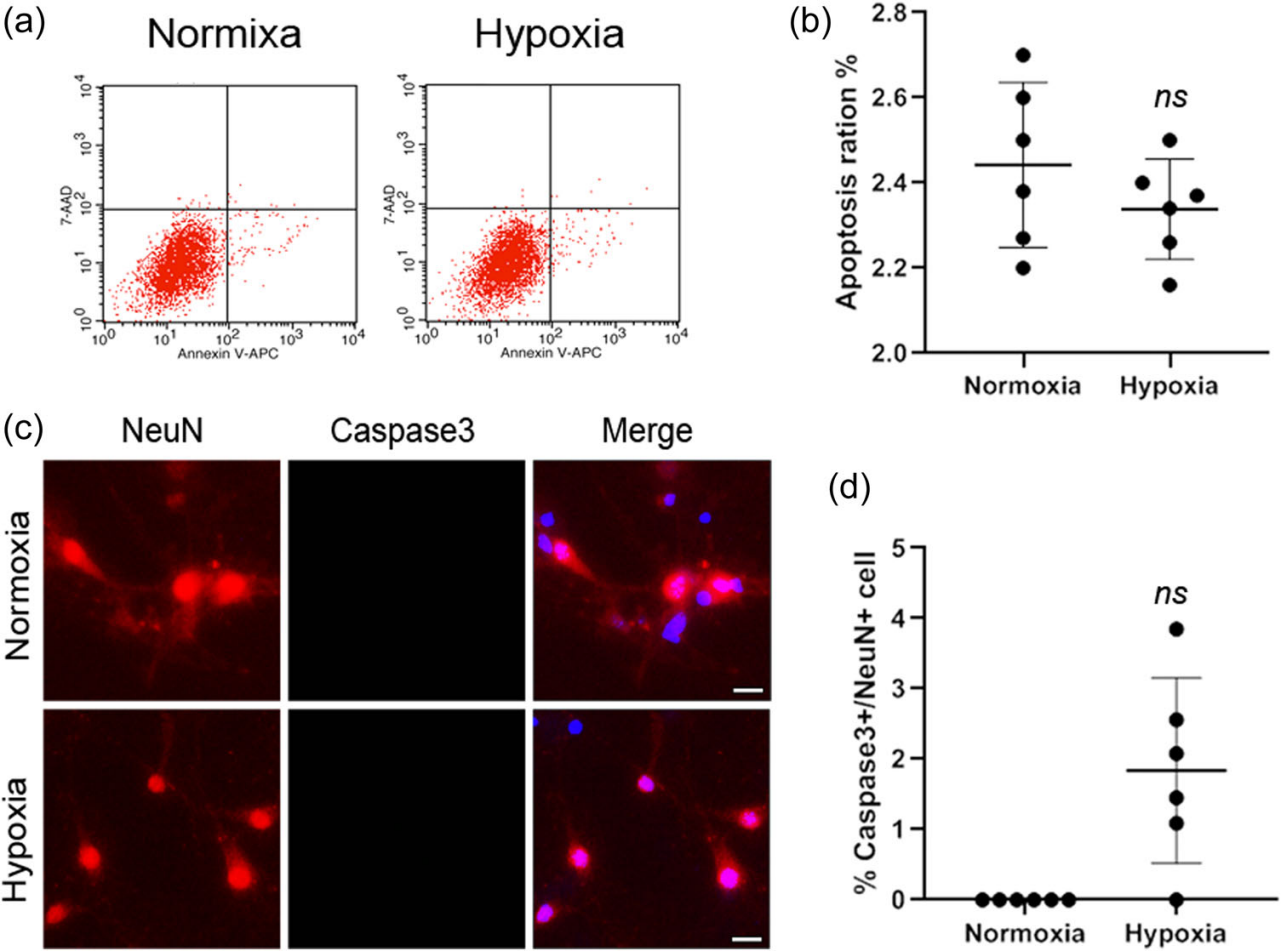
Cell apoptosis evaluation of NMR hippocampal neurons. (a) Apoptosis of NMR hippocampal neurons under normoxic or hypoxic conditions was evaluated by flow cytometry. (b) Statistical analysis of apoptosis levels of NMR hippocampal neurons (*n* = 6 per group). (c) Apoptosis of mouse hippocampal neurons under normoxic or hypoxic conditions was evaluated by immunocytochemistry (scale bar: 20 μm). (d) Statistical analysis of apoptosis levels of NMR hippocampal neurons. Data are shown as the means ± *SEM*. Experiments were repeated for six times. NMR, naked mole‐rats

We first characterised the expression pattern of Cav‐1 in NMR hippocampal neurons. The results (Figure 1a,b) showed that HIF1‐α levels were upregulated in NMR hippocampal neurons, especially 8 h after hypoxia treatment, which correlated with the pattern of Cav‐1 expression. In addition, results (Figure 1a and 1c) showed that when NMR hippocampal neurons were subjected to hypoxic conditions (O_2_, 8%) for 0–12 h, the level of Cav‐1 increased and peaked at 8 h of hypoxia treatment. In the meanwhile, Caspase3 protein levels decreased significantly 8 h after hypoxia (Figure 1a and 1d). Immunochemistry (Figure 1e,f) revealed that immunofluorescence intensity of Cav‐1 in NMR hippocampal neurons increased significantly under conditions of hypoxia (8% O_2_) for 8 h versus normoxic conditions (20% O_2_), which was also quantified by immunointensity analysis (Figure 1f). Flow cytometry (Figure 2a,b) and immunocytochemistry (Figure 2c,d) showed that during hypoxia, there was no significant increase in the apoptosis of NMR hippocampal neurons.

### 3.2 Cav‐1 reduces the death of NMR hippocampal neurons under hypoxia in vitro

**Figure 3:**
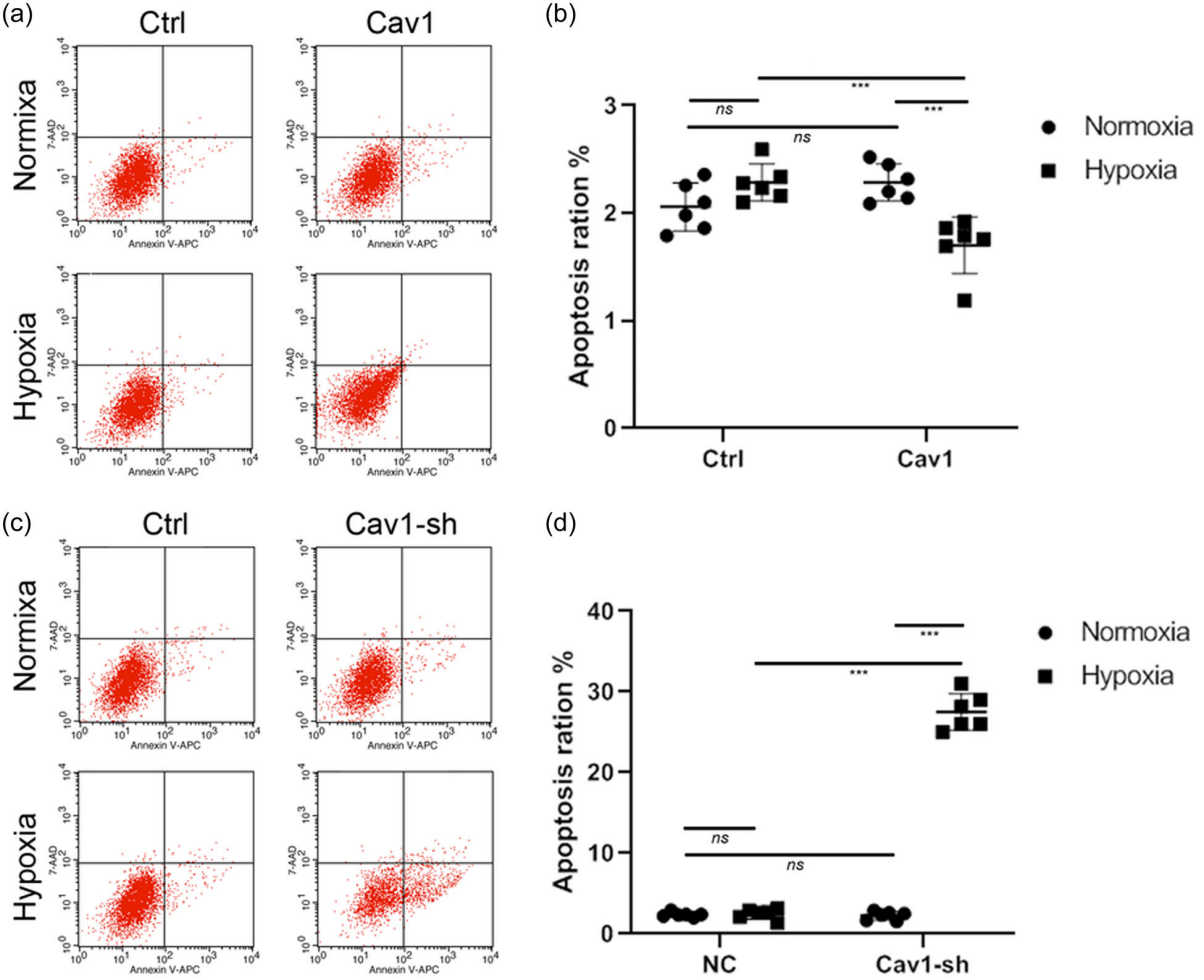
Exploring the roles of Cav‐1 in regulating the apoptosis of naked mole rat hippocampal neurons by flow cytometry. (a) Apoptosis of NMR hippocampal neurons transfected with LV‐GFP (ctrl) or NMR origin LV‐Cav‐1 (Cav‐1) under normoxic or hypoxic conditions was evaluated by means of flow cytometry. (b) Statistical analysis of apoptosis levels of NMR hippocampal neurons. (c) Apoptosis of NMR hippocampal neurons transfected with LV‐scramble RNA (ctrl) or shRNA against NMR Cav‐1(Cav1‐sh) under normoxic or hypoxic conditions was evaluated by means of flow cytometry. (d) Statistical analysis of apoptosis levels of NMR hippocampal neurons. Data are presented as the mean ± *SEM*. Experiments were repeated for 6 times. ***p < .001, *ns*, no significant differences. NMR, naked mole‐rats; shRNA, short hairpin RNA

To more specifically assess the influence of Cav‐1 on the survival of neurons from NMRs during hypoxia, Cav‐1 was upregulated or downregulated by transfecting LV‐Cav‐1 or LV‐sh‐Cav‐1 into cultured NMR hippocampal neurons. Flow cytometry analysis showed that Cav‐1 overexpression (Figure 3a,b) did not increase the apoptosis levels of NMR hippocampal neurons during normoxia. But Cav‐1 overexpression led to the reduction of NMR hippocampal neuron apoptosis levels. However, downregulation of Cav‐1 by LV‐sh‐Cav‐1 (Figure 3c,d) led to a significant increase in hippocampal neuron apoptosis.

### 3.3 Cav‐1 reduces the apoptosis of NMR hippocampal neurons under hypoxia via HIF1‐α in vitro

**Figure 4:**
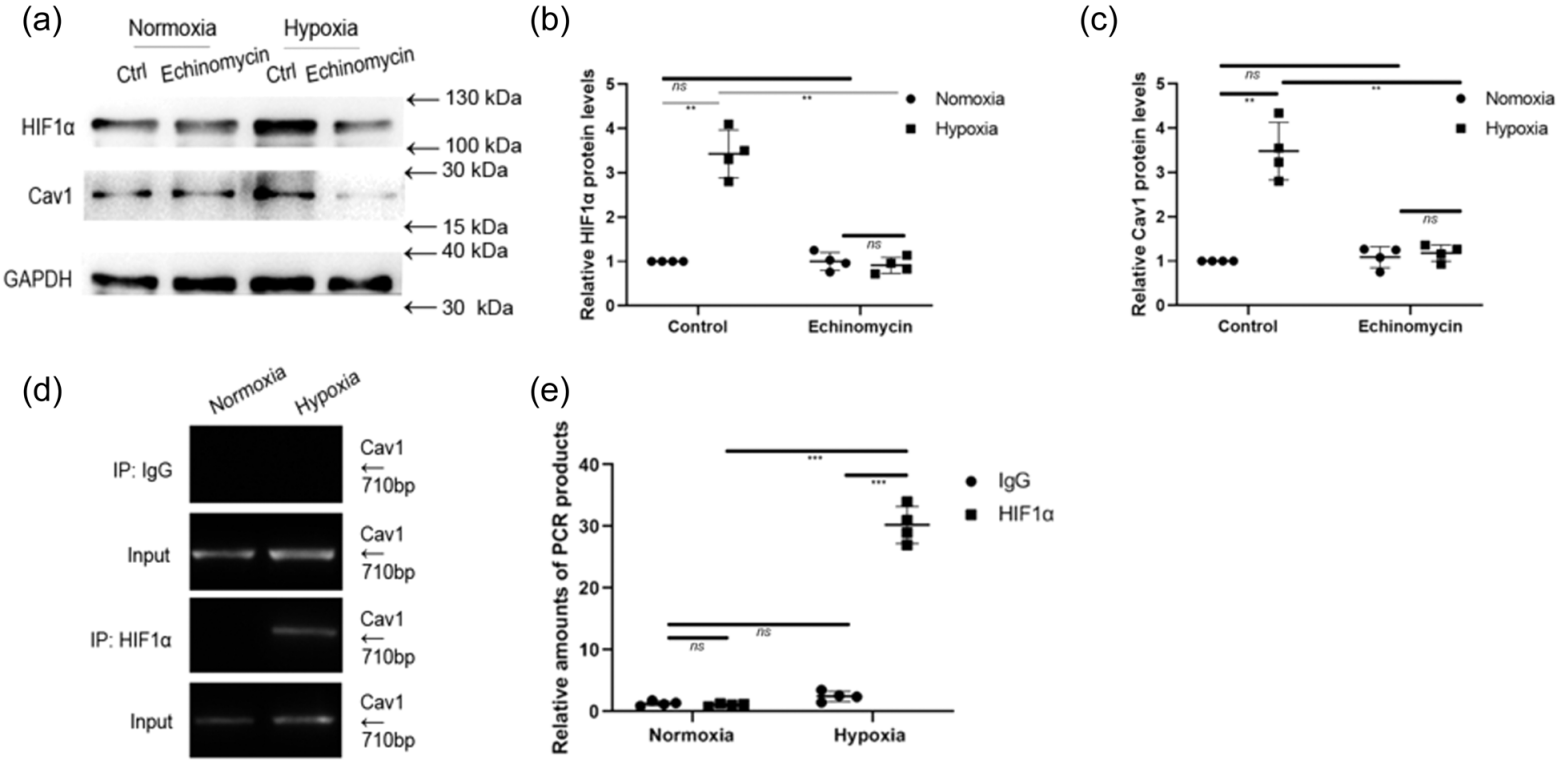
HIF1α regulates the levels of Cav‐1. (a) Primary NMR hippocampal neurons were incubated with or without 20 mM echinomycin under normoxia or hypoxia (8% O_2_) for 8 h with HIF1α and Cav‐1. (b, c) Statistical analysis of relative HIF1α or Cav‐1 protein levels. The results are shown as the mean ± *SEM* (*n* = 4 per group). (d) Hippocampal neurons were incubated under hypoxia or normoxia for 8 h, and ChIP was performed with anti‐HIF‐1α or control IgG. (e) The results are shown as the mean ± *SEM*. Experiments were repeated for four times. **p < .01, ***p < .001, *ns*, no significant differences. HIF, hypoxia inducible factor‐1α; NMR, naked mole‐rats; IgG, immunoglobulin G

HIF‐1 is a master transcriptional regulator of numerous genes under hypoxic conditions. To examine whether the regulatory role of HIF‐1α in the expression levels of caveolin‐1 was investigated. Echinomycin was used to inhibit the binding of HIF‐1α to hypoxia response elements (HREs) in the DNA. Western blotting analysis showed that the inhibition of HIF‐1α by echinomycin (Figure 4a,b) restored the expression levels observed under normoxia. In the meanwhile, Cav‐1 protein levels were also decreased significantly (Figure 4a and 4c).

Furthermore, the relationship between HIF1α and Cav‐1 were investigated by ChIP assays, which were performed by using an anti‐HIF‐1α antibody and Cav‐1 promoter elements containing putative HIF‐responsive element (HRE) consensus sequences. HIF‐1α binds to HRE‐containing Cav‐1 promoter elements (Figure 4d,e) in cultured hippocampal neurons under hypoxia.

**Figure 5:**
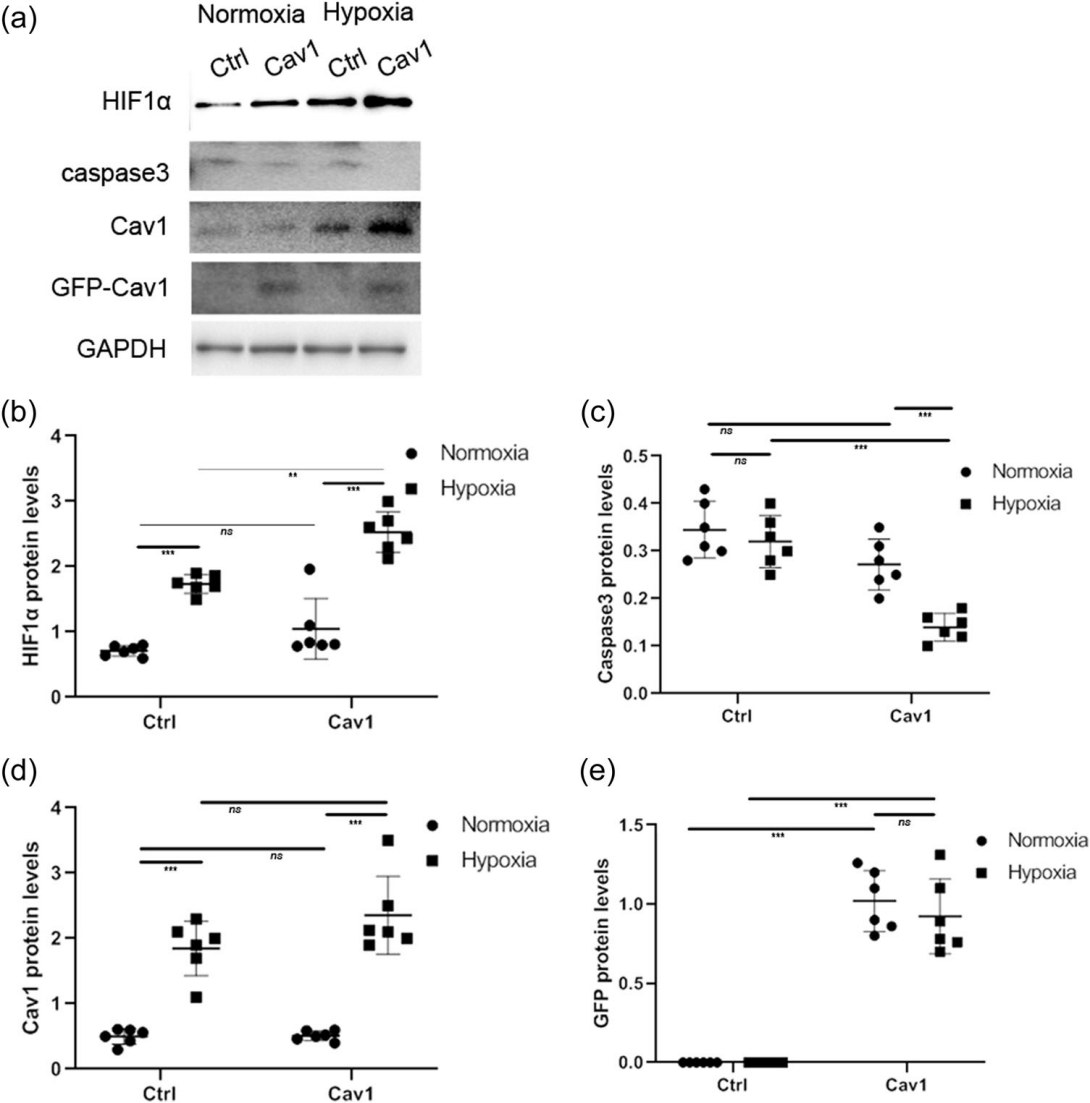
Exploring the roles of Cav‐1 in regulating the apoptosis of naked mole rat hippocampal neurons by Cav‐1 overexpression. (a) Primary NMR hippocampal neurons transfected with LV‐GFP (ctrl) or shRNA against NMR origin LV‐Cav‐1 (Cav‐1) were incubated under hypoxia or normoxia for 8 h, and the levels of HIF‐1α, caspase3 and Cav‐1 were examined. (b–d) The relative protein levels of caspase3, HIF‐1α and Cav‐1 under hypoxia or normoxia are shown as the mean ± *SEM*. (e) The relative protein levels of GFP under hypoxia or normoxia are shown as the mean ± *SEM*. Experiments were repeated for six times. **p < .01, ***p < .001, *ns*, no significant differences. NMR, naked mole‐rats; shRNA, short hairpin RNA

**Figure 6:**
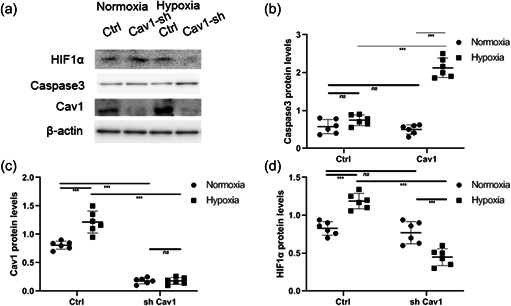
Exploring the roles of Cav‐1 in regulating the apoptosis of naked mole rat hippocampal neurons by knocking down Cav‐1. (a) Primary NMR hippocampal neurons transfected with LV‐scramble RNA (ctrl) or shRNA against NMR origin LV‐Cav‐1 (Cav1‐sh) were incubated under hypoxia or normoxia for 8 h, and the levels of HIF‐1α, caspase3 and Cav‐1 were examined. (b–c) The relative protein levels of caspase3, and Cav‐1 under hypoxia or normoxia are shown as the mean ± *SEM*. Experiments were repeated for six times. ***p < .001, *ns*, no significant differences. NMR, naked mole‐rats; shRNA, short hairpin RNA

Western blotting analysis showed that both HIF‐1α and Cav‐1 levels increased (Figure 5a,b and 5d) significantly in the Cav‐1 overexpression group versus the control during hypoxia. Cav‐1 overexpression decreased caspase‐3 levels (Figure 5c) during hypoxia in NMR hippocampal neurons compared with normoxia. In addition, the levels of transfected Cav‐1 carrying GFP (Figure 5a and 5e) were verified by western blotting analysis. Caspase‐3 levels (Figure 6a,b) increased significantly compared with the control group during hypoxia. In addition, the levels of Cav‐1 and HIF‐α (Figure 6a–d) were verified by western blotting analysis.

### 3.4 Cav‐1 reduces the apoptosis of neurons in vivo during hypoxia

**Figure 7:**
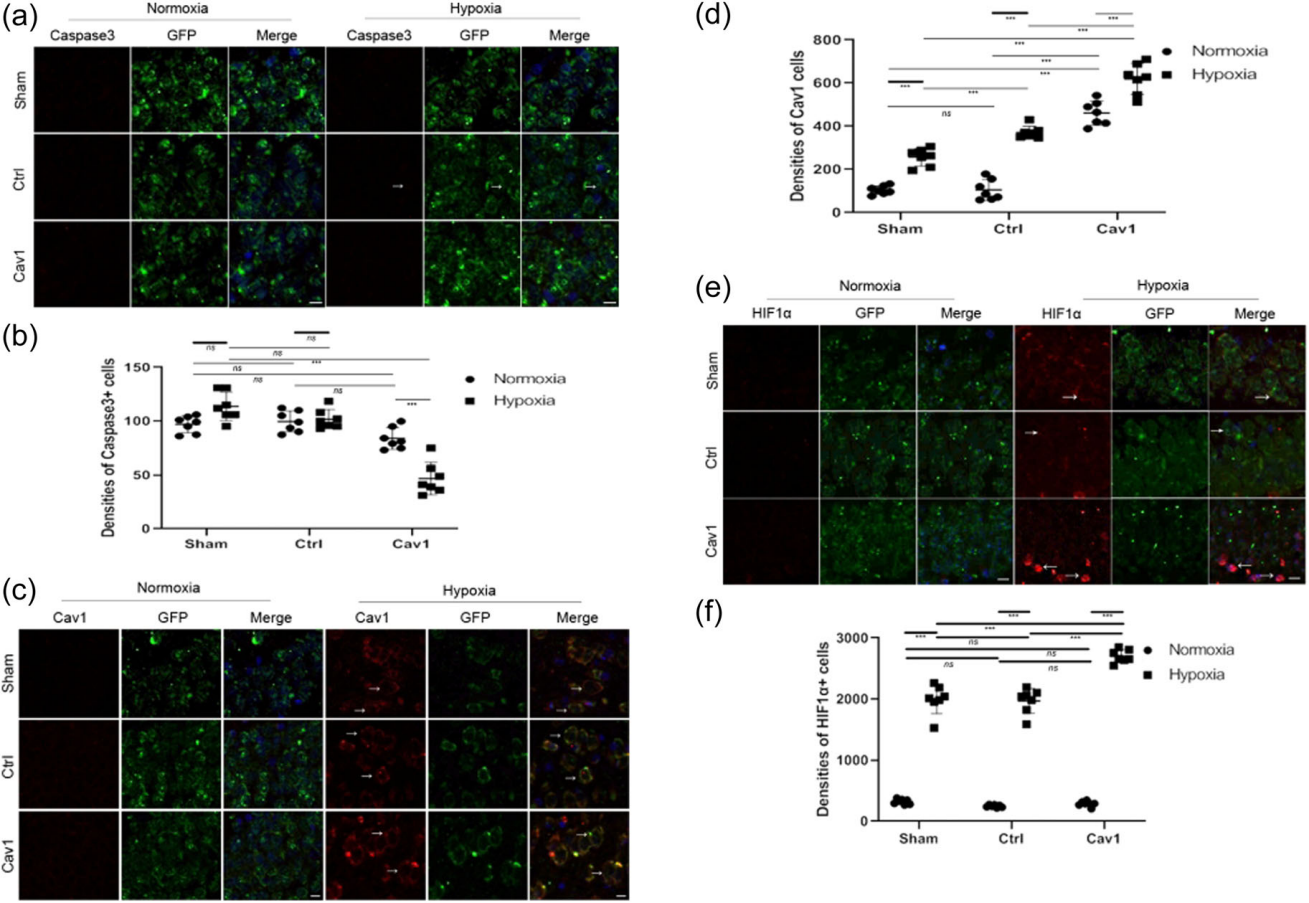
Roles of Cav‐1 in regulating the apoptosis of naked mole rat hippocampal neurons in Cav‐1 overexpression NMR hypoxia models. (a) NMRs transfected with NMR origin Cav‐1 or LV‐GFP (ctrl) were maintained under hypoxia or normoxia for 8 h, and caspase‐3 levels in hippocampal neurons were then examined by immunohistochemistry. (b) The density of caspase‐3^+^ hippocampal neurons under hypoxia or normoxia was evaluated. (c) NMR‐transfected NMR origin Cav‐1 or ctrl vectors (LV‐GFP) were maintained under hypoxia or normoxia for 8 h, and Cav‐1 levels in hippocampal neurons were then examined by immunohistochemistry. (d) The density of Cav‐1^+^ hippocampal neurons under hypoxia or normoxia was evaluated. (e) NMRs transfected with NMR origin Cav‐1 or LV‐GFP (ctrl) were maintained in hypoxia or normoxia for 8 h, and HIF‐1α levels in hippocampal neurons were then examined by immunohistochemistry. (f) The density of HIF‐1α^+^ hippocampal neurons under hypoxia or normoxia was evaluated (scale bar: 10 μm). The results are shown as the mean ± *SEM* (*n* = 7 animals per group). ***p < .001, *ns*, no significant differences. NMR, naked mole‐rats

Given that Cav‐1 is upregulated in hippocampal neurons subjected to hypoxia, we explored the role of hypoxia‐induced Cav‐1 in the NMR brain after hypoxia. We investigated the role of Cav‐1 in NMRs after 8 h of hypoxia treatment (O_2_, 8%). Immunohistochemistry analysis revealed no significant differences in the density of caspase3^+^/GFP^+^ hippocampal neurons (Figure 7a,b) between the sham group and the control group under conditions of hypoxia versus normoxia group. But after transfection with Cav‐1, the density of caspase3^+^/GFP^+^ hippocampal neurons decreased significantly in hypoxia conditions compared with those in normoxia conditions. The densities of Cav‐1^+^ cells in the ctrl and Cav‐1 overexpression groups (Figure 7c,d) were increased under hypoxic conditions versus those in the normoxia group. The density of HIF1α^+^/GFP^+^ hippocampal neurons (Figure 7e,f) in the Cav‐1 overexpression group during hypoxia treatment was significantly higher than that under normoxic conditions.

**Figure 8:**
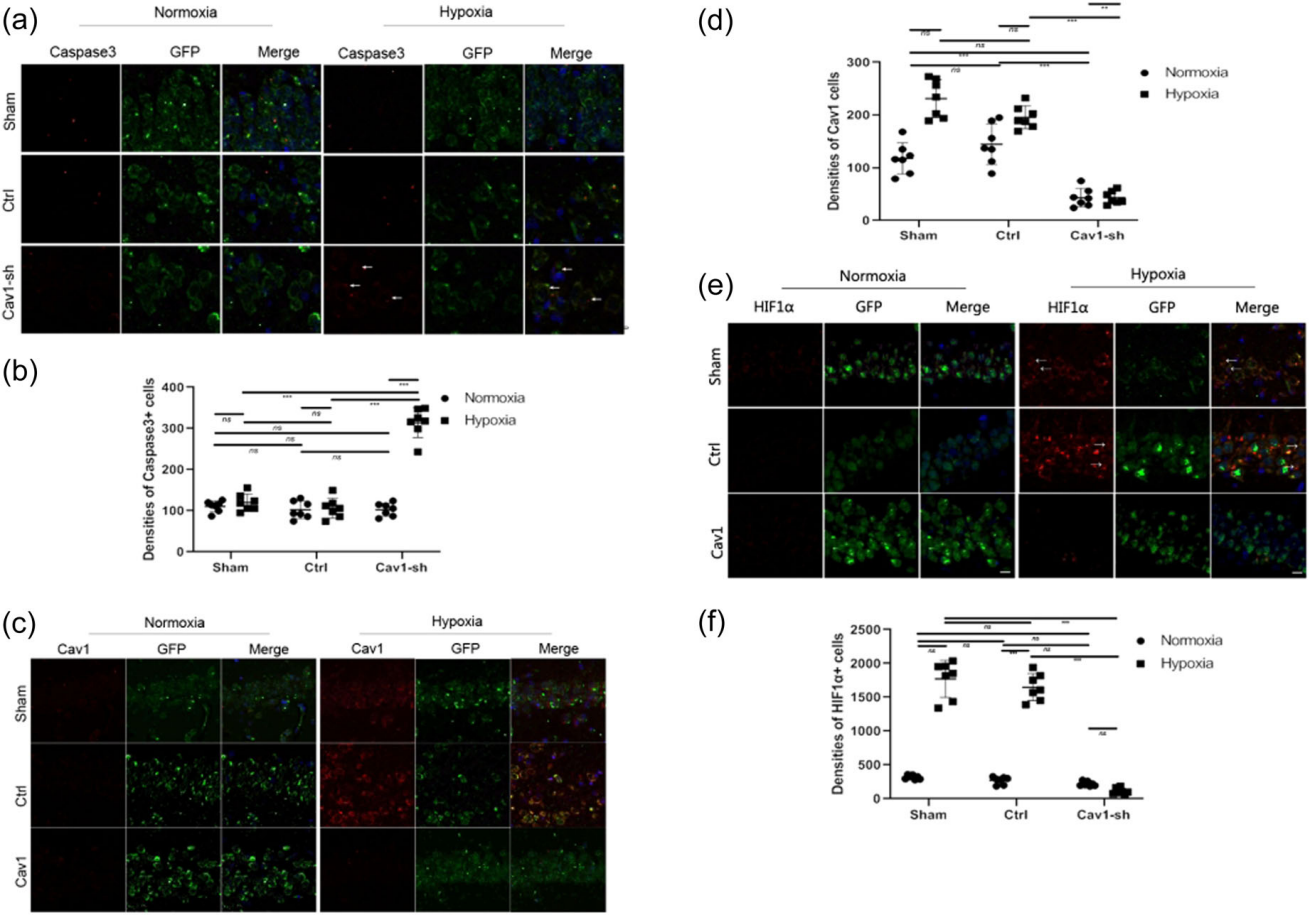
Roles of Cav‐1 in regulating the apoptosis of naked mole rat hippocampal neurons in Cav‐1 downregulation NMR hypoxia models. (a) NMR‐transfected NMR‐sh‐Cav‐1 or LV‐scramble RNA (ctrl) were kept under hypoxia or normoxia for 8 h, and caspase‐3 levels in hippocampal neurons were then examined by immunohistochemistry. (b) The density of caspase3^+^ hippocampal neurons under hypoxia or normoxia was evaluated. (c) NMR‐transfected NMR‐sh‐Cav‐1 or ctrl vectors were maintained under hypoxia or normoxia for 8 h, and Cav‐1 levels in hippocampal neurons were then examined by immunohistochemistry. (d) The density of Cav‐1^+^ hippocampal neurons under hypoxia or normoxia was evaluated. (e) NMR spectra following transfection with NMR‐Cav‐1‐sh or ctrl vectors were maintained under hypoxia or normoxia for 8 h, and HIF‐1α levels in hippocampal neurons were then examined by immunohistochemistry. (f) The density of HIF‐1α^+^ hippocampal neurons under hypoxia or normoxia was evaluated (scale bars: 10 μm) The results are presented as the mean ± *SEM* (*n* = 7 animals per group). **p < .01, ***p < .001, *ns*, no significant differences. NMR, naked mole‐rats

In addition, we investigated the hypoxia tolerance of NMR hippocampal neurons when Cav‐1 was downregulated during hypoxia. Immunohistochemistry analysis showed that the density of caspase3^+^/GFP^+^ hippocampal neurons (Figure 8a,b) in the LV‐sh‐Cav‐1 group during hypoxia treatment was much higher than that under normoxic conditions. Immunohistochemistry analysis also showed significant downregulation of Cav‐1^+^/GFP^+^ cell density (Figure 8c,d) in the LV‐sh‐Cav‐1 group. In addition, the densities of HIF1α^+^/GFP^+^ cells (Figure 8e,f) decreased significantly compared with those of the ctrl group during hypoxia.

We also investigated the role of Cav‐1 in regulating hypoxia tolerance in mice. Immunohistochemistry analysis showed that when Cav‐1 was transfected into the mouse hippocampus, the density of HIF1α^+^/GFP^+^ hippocampal neurons during hypoxia treatment significantly increased compared with Ctrl groups under hypoxia (Figure S2a,b). In the meanwhile, the density of Caspase3^+^/GFP^+^ hippocampal neurons under hypoxic treatment was less than those under normoxia (Figure S2e,f). Cav‐1 levels also increased significantly (Figure S2c,d). When Cav‐1 was knocked down by sh‐Cav‐1 within hippocampus, the density of HIF1α^+^/GFP^+^ hippocampal neurons during hypoxia treatment significantly decreased compared with Ctrl groups under hypoxia (Figure S3a–d). In the meanwhile, the density of Caspase3^+^/GFP^+^ hippocampal neurons under hypoxic treatment was much more than those under normoxia (Figure S3e,f).

**Figure 9:**
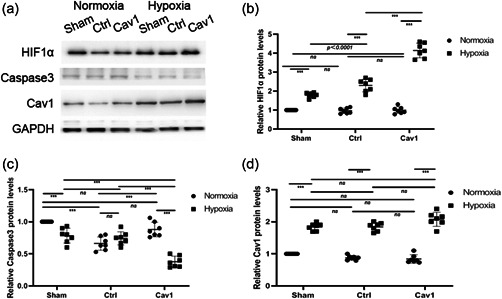
Roles of Cav‐1 in regulating NMR hippocampal neuron apoptosis levels analysed by western blotting in NMR hypoxia models. (a) NMR‐transfected NMR‐Cav‐1 or LV‐GFP (ctrl) were maintained under hypoxia or normoxia for 8 h, and hippocampal neurons were subsequently examined by immunohistochemistry using anti‐HIF‐1α, Cav‐1, and caspase3 antibodies. (b–d) The relative protein levels of HIF1α, caspase3 and Cav‐1 under hypoxia or normoxia were evaluated. The results are presented as the mean ± *SEM* (*n* = 7 animals per group). ***p < .001, *ns*, no significant differences. HIF, hypoxia inducible factor‐1α; NMR, naked mole‐rats

**Figure 10:**
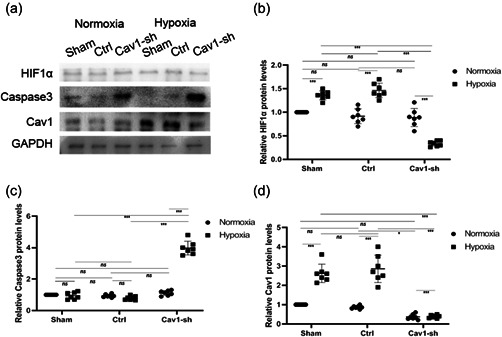
Roles of Cav‐1 in regulating NMR hippocampal neuron apoptosis levels analysed by western blotting in NMR hypoxic models. (a) NMR‐transfected LV‐Cav1 or LV‐GFP (ctrl) cells were maintained under hypoxia or normoxia for 8 h, and hippocampal neurons were subsequently examined by immunohistochemistry using anti‐caspase3, HIF‐1α, and Cav‐1 antibodies. (b–d) The relative protein levels of HIF1α, caspase3 and Cav‐1 under hypoxia or normoxia were evaluated. The results are presented as the mean ± *SEM* (*n* = 7 animals per group). *p < .05, **p < .01, ***p < .001, *ns*, no significant differences. HIF, hypoxia inducible factor‐1α; NMR, naked mole‐rats

Furthermore, western blotting analysis was performed to assess caspase‐3 and HIF‐1α levels in hypoxia‐NMR models in vivo. When Cav‐1 protein levels were overexpressed, HIF‐1α (Figure 9a,b) levels increased significantly after hypoxia treatment in every group. Caspase3 levels (Figure 9a and 9c) decreased significantly after hypoxia treatment. In addition, Cav‐1 protein levels (Figure 9a and 9d) were also verified by western blotting analysis. When Cav‐1 levels were downregulated, HIF‐1α levels (Figure 10a,b) decreased dramatically versus normoxia. Caspase3 levels (Figure 10d,e) increased significantly after hypoxia treatment. Cav‐1 levels (Figure 10d and 10g) were also evaluated by western blotting analysis.

In Cav‐1 overexpression mice, there is the increase in HIF‐1α and Cav‐1 levels under hypoxic conditions, whereas Caspase‐3 levels did not increase significantly during hypoxia (Figure S4a–e). In Cav‐1 knocked down mice, the levels of HIF1α and Cav‐1 decreased and the Caspase3 protein levels increased significantly.

## 4 DISCUSSION

Human beings are always faced with a variety of hypoxia threats, such as pathological conditions including cancer, diabetes, aging, and low‐oxygen environments such as the deep sea and space (Chervona & Costa, 2012; Hermand et al., 2017). The mammalian brain is one of the highest oxygen consumption organs, accounting for approximately 20% of body energy, and is extremely sensitive to the lack of oxygen and nutrition (Kim et al., 2014; Wong et al., 2013; Yoon et al., 2015). In pathological conditions, a lack of oxygen and/or nutrients for a few minutes may lead to permanent neuronal death, or even related dysfunctions (Toman et al., 2019). NMRs survive in acute or chronic hypoxia for a long time (Larson et al., 2014, Larson & Park, 2009), but they maintain normal life activities and a healthy state, especially neural cells and brain functions are unaffected. The changes in adaptation to hypoxic environments that occurred during its evolution are advantages that other rodents that adapt to normoxic environments do not exhibit, and will be of great value for developing preventive and therapeutic strategies for ischaemic and anoxic diseases occurring in the nervous system, such as ischaemic stroke.

This study aims to explore the mechanisms for brain hypoxic tolerance in NMRs and clarify the variance between NMRs and mammals adapted to normoxia to find effective ways that may be useful in preventing brains from hypoxic impairment during pathological conditions. The potential role of Cav‐1 in hypoxia‐induced injury in NMR hippocampal neurons has been investigated, producing the following conclusions. First, Cav‐1 expression was upregulated in NMR hippocampal neurons after hypoxia. Second, Cav‐1 exerted a protective effect in NMR hippocampal neurons subjected to hypoxia. Inhibition of Cav‐1 by lentiviral vectors aggravated NMR hippocampal neuron damage. Increased Cav‐1 expression using LV‐Cav‐1 led to significantly increased hypoxia tolerance in NMR hippocampal neurons. Finally, HIF‐1α modulated Cav‐1 expression in NMR hippocampal neurons under hypoxic conditions. In vitro experiments with hippocampal slices showed that, compared with mice, NMRs survived acute hypoxic conditions, including anaerobic conditions, and neurons died little (Larson et al., 2014). In addition, sustained upregulation on GluN2D subunit of NMDA receptors (Peterson et al., 2012) may be one of the reasons for their brain hypoxia tolerance as observed in mammalian neonates (Bickler, 2004). In vivo experiments have demonstrated that intact NMRs tolerate severe hypoxia (5% O_2_) and anoxia much longer than mice (Park et al. 2017). By utilising fructose as the energy supplier independent of oxygen, NMRs tolerate anoxia for 18 min, approximately 18 times longer than mice. These findings strongly indicate the extreme hypoxia tolerance of NMRs, and the underlying mechanisms urgently need to be explored.

Cav‐1 is an essential structural protein of caveolae that is involved in many cellular events and plays as a key regulator under ischemia (Xu et al., 2015). The expression level of Cav‐1 after ischemic injury in mammalian brain still remains controversial (Huang et al., 2012; Jasmin et al., 2007; Mao et al., 2015). Cav‐1 promotes the survival of the neuronal cell line (neuro‐2a) in mice deprived of glucose and oxygen (Zhong et al., 2020). However, after glucose and oxygen deprivation, the expression of Cav‐1 in astrocytes decreased significantly (Xu et al., 2016). This may be related to the different responses of neurons and glial cells to hypoxia. Despite differences in Cav‐1 levels after hypoxia, a protective role of Cav‐1 in the CNS has been demonstrated during hypoxia. Currently, there were no studies focused on Cav‐1 function have been reported in NMRs, a naturally hypoxia‐tolerant animal. In this study, it has been found that Cav‐1 was increased in NMR hippocampal neurons following hypoxia treatment in hypoxic models in vitro and in vivo. Similarly, it has also been reported that Cav‐1 levels increase in the spinal cord after ischaemia‐reperfusion injury (Yang et al., 2016). Cav‐1 KO mice exhibited a larger lesion volume and worse behavioural outcomes than wild‐type mice after ischaemic injuries (Jasmin et al., 2007) and increased BBB permeability (Gu et al., 2012). These results suggest the protective role of Cav‐1 in central nervous system ischaemia diseases. In particular, the upregulation of Cav‐1 in NMR hypoxic cellular and animal models strongly indicates that it is one of the most important potential molecules regulating NMR hypoxia tolerance and may be a profound candidate in the prevention of hypoxic diseases.

HIF‐1α plays an important role in the mammalian cells responding to hypoxia (Huang et al., 1998), whose levels are precisely regulated by oxygen concentration and other mechanisms (Jain et al., 2016). Furthermore, HIF‐1α has been reported to play a key role in regulating the final outcome of hypoxic diseases such as ischaemic stroke (Abdullahi et al., 2020; Koh et al., 2015; Shi, 2009). Whether HIF‐1α is involved in the regulation of hypoxia tolerance in the NMR brain is unclear. Kim (2011) and colleagues found that NMR HIF‐1α revealed a T407I mutation that is different from other mammals and is localised in the VHL‐binding domain, which may be responsible for avoiding ubiquitin‐dependent degradation of HIF‐1α, but they did not show direct evidence of HIF‐1α in regulating NMR hypoxia adaptation. In this study, both Cav‐1 and HIF‐1α levels increased significantly in NMR hippocampal neurons after hypoxia treatment. In the context of Cav‐1 overexpression, NMR hippocampal neurons may increase hypoxia tolerance. ChIP analysis (Figure 4a–c) showed that the levels of Cav‐1 were regulated by HIF‐1α under hypoxia, indicating the relationships between HIF‐1α and Cav1. When the HIF‐1α levels were furtherly inhibited by echinomycin, Cav‐1 levels were significantly downregulated, which strongly shows that Cav‐1 levels were regulated by HIF‐1α under hypoxia. Besides, downregulation of Cav‐1 in vitro did not lead to the reduction of HIF1α as shown in Figure 6, which provided a second proof for the regulation of HIF‐1α in regulating the Cav‐1 levels. However, Yue and colleagues found that Cav‐1 is a neuronal receptor for the intake of EC‐derived EVs and is upregulated in neurons by oxygen and glucose deprivation stimulation (Yue et al., 2019), thus resulting in much more exosomal intake by neurons. These results indicate that certain stresses, including hypoxia, may evoke the tolerance of cells to their microenvironment (Yue et al., 2019). Based on our observation that the Cav‐1 protein level is regulated during hypoxia, we wondered whether HIF‐1α was able to modulate Cav‐1 expression in NMR hippocampal neurons after hypoxia simulation. ChIP assays were performed to demonstrate that HIF‐1α binds to the Cav‐1 promoter under hypoxic brain conditions (8% O_2_). Taken together, these results indicated that Cav‐1 is a downstream effector of HIF‐1α during hypoxia. Our data suggest a more comprehensive mechanism of Cav‐1‐ and HIF‐1α‐mediated neuronal protection during hypoxia. Thus, HIF1‐α plays a neuronal protective role after the induction of Cav‐1, which may mediate exosomal intake in hypoxia, as previously reported. But during hypoxia, Cav‐1 knocking down is accompanied by the downregulation of HIF‐1α. The mechanisms behand this phenomenon remains unclarified. But this phenomenon maybe supported by some related researches as follows. It has been found that Cav‐1 is a central integrator of multiple signals including tyrosine kinase, the EGFR family and nuclear factor kappa B (NF‐κB) (Kim & Hirabayashi, 2018; Ma et al., 2016; Wang et al., 2011), which can be used as an upstream regulator of NF‐κB (Azoitei et al., 2016; Jiao et al., 2019). In some conditions, HIF‐1α is regulated by NF‐κB (Ahmmed et al., 2019). Besides, the activation of NF‐κB by HIF‐1α, the transcriptional activation of HIF‐1α by NF‐κB may occur simultaneously (Azoitei et al., 2016). Thus, we speculate that the change of Cav‐1 levels may indirectly affect the levels of HIF‐1α. These findings further indicate the profound roles of Cav‐1 in the prevention and treatment of hypoxic‐ischaemic brain diseases.

To determine the role of Cav‐1 in contributing to the pathogenesis of brain ischaemia, Cav‐1 levels were regulated by lentiviral vectors in vivo and in vitro following hypoxia treatment. Notably, we found that Cav‐1 overexpression increased the survival of NMR neuronal cells within the hippocampus following hypoxia. In contrast, knocking down Cav‐1 led to the death of neuronal cells within the hippocampus on a large scale. Although there were neurons, glial cells, endothelial cells, etc., in the hippocampus, the percentage of neurons was constant. Therefore, the percentage of hippocampal neurons transfected with Cav‐1 was similar. These results demonstrated the pivotal role of Cav‐1 during NMR hypoxia tolerance in vivo. Furthermore, it is still interesting to investigate the role of Cav‐1 in hippocampal neurons by specifically overexpressing or knocking down Cav‐1 in NMR neurons in vivo. Previous investigations have demonstrated that Cav‐1 is associated with several central nervous diseases. Choi et al. (2016) have reported that overexpression of Cav‐1 attenuates brain oedema by inhibiting tight junction degradation, suggesting a protective role for Cav‐1 in brain ischaemic disease. Our findings, taken together with these previous reports, suggest that Cav‐1 may be closely associated with the pathogenesis of CNS hypoxic diseases.

In this study, Cav‐1 was found to be a pivotal molecular player regulating hypoxia tolerance in NMR hippocampal neurons. We reveal that Cav‐1 is upregulated in a HIF‐1α‐dependent manner during hypoxia and that Cav‐1 overexpression significantly reduces hippocampal neuron apoptosis during hypoxia in vivo and in vitro. Downregulation of Cav‐1 increased the apoptosis of NMR hippocampal neurons during hypoxia. In addition, the upregulation of Cav‐1 during hypoxia was regulated by HIF‐1α, which was verified by ChIP analysis. Thus, further investigation of the functions of Cav‐1 and HIF‐1α during CNS hypoxia may provide invaluable solutions for the development of effective therapies against hypoxia‐related CNS diseases.

## 5 CONCLUSIONS

The potential role of Cav‐1 in hypoxia‐induced injury in NMR hippocampal neurons has been investigated, producing the following conclusions. First, Cav‐1 expression was upregulated in NMR hippocampal neurons after hypoxia. Second, Cav‐1 exerted a protective effect in NMR hippocampal neurons subjected to hypoxia. Inhibition of Cav‐1 by lentiviral vectors aggravated NMR hippocampal neuron damage. Increased Cav‐1 expression using LV‐Cav‐1 led to significantly increased hypoxia tolerance in NMR hippocampal neurons. Finally, HIF‐1α modulated Cav‐1 expression in NMR hippocampal neurons under hypoxic conditions.

## AUTHOR CONTRIBUTIONS

**Wenjing Yang**: conceptualisation, data curation, formal analysis, methodology, project administration, supervision, validation, visualisation, writing—original draft; writing—review and editing. **Wenqing Wu**: data curation, formal analysis, methodology, and validation. **Ying Zhao**: data curation and formal analysis. **Yu Li**: methodology. **Chengcai Zhang**: formal analysis and investigation. **Jingyuan Zhang**: investigation and methodology. **Chao Chen**: investigation and methodology. **Shufang Cui**: formal analysis, methodology, project administration, resources, and supervision.

## CONFLICT OF INTEREST

The authors declare no conflict of interest.

## Supporting information

Supporting information.

## Data Availability

The data that support the findings of this study are available from the corresponding author upon reasonable request. The datasets used and analyzed in the current study are available from the corresponding author upon reasonable request.
