# Supplementary material for: Caveolin‐1 suppresses hippocampal neuron apoptosis via the regulation of HIF1α in hypoxia in naked mole‐rats
Source: Cell Biol Int. 2022 Sep 2;46(12):2060–74. doi: 10.1002/cbin.11890 (PMC9826031; doi:10.1002/cbin.11890)
Supplement: Supplementary file 1 — Supporting information. [file CBIN-46-2060-s001.doc]

**Supplementary Material**


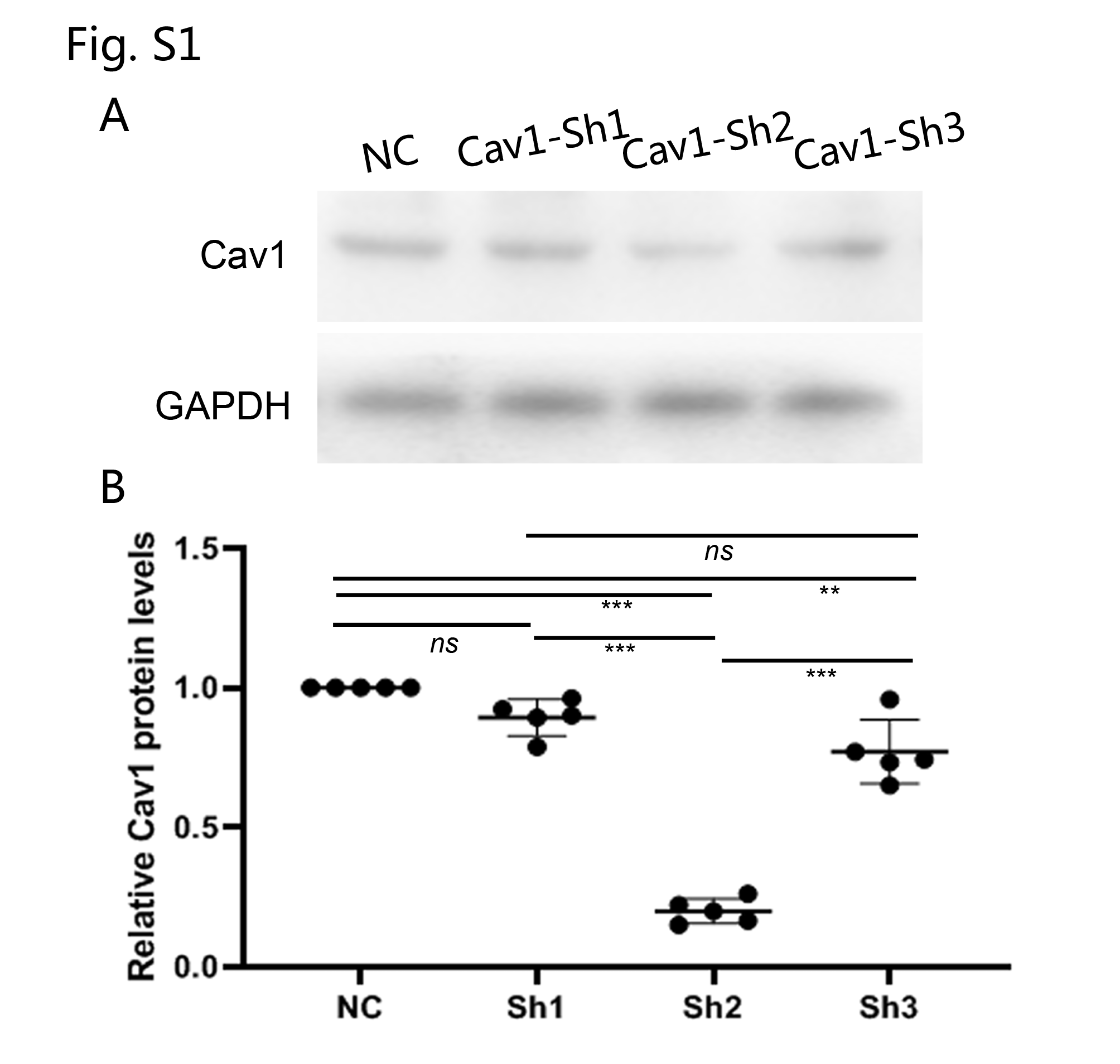


FIGURE S1 Verification of the shRNAs against NMR Cav-1 and determination of the levels of Cav-1 in mouse hippocampal neurons Cav-1 during hypoxia and normoxia. (A) Western blotting analysis of the efficiency of three shRNAs against NMR Cav-1. (B) Statistical analysis of protein levels investigated by western blotting analysis. The results are shown as the mean ± SEM. Experiments were repeated for 4 times. ** *p*＜0.01, *** *p*＜0.001, ns, no significant differences.


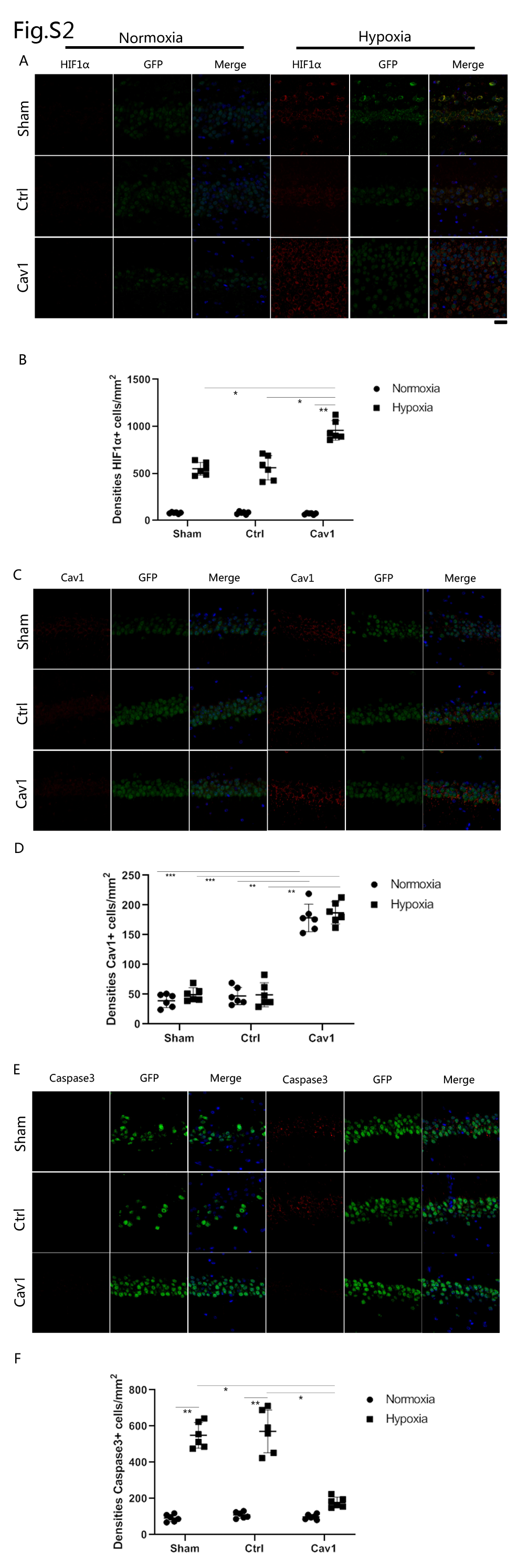


FIGURE S2 Roles of Cav-1 in regulating the apoptosis of mice hippocampal neurons in Cav-1 overexpression mice hypoxia models. (A) Mice transfected with Cav-1 or LV-GFP (ctrl) were maintained under hypoxia or normoxia for 8 h, and HIF-1α levels in hippocampal neurons were then examined by immunohistochemistry. (B) The density of HIF-1α^+^/GFP^+^cells under hypoxia or normoxia was evaluated. (Scale bar, 10 μm). The results are shown as the mean ± SEM (n=6 animals per group). (C) Mice transfected with Cav-1 or LV-GFP (ctrl) were maintained under hypoxia or normoxia for 8 h, and Cav-1 levels in hippocampal neurons were then examined by immunohistochemistry. (D) The density of Cav-1^+^/GFP^+^ cells under hypoxia or normoxia was evaluated. (E) Mice transfected with Cav-1 or LV-GFP (ctrl) were maintained under hypoxia (10% O2, the same as follows) or normoxia for 8 h, and caspase3 levels in hippocampal neurons were then examined by immunohistochemistry. (F) The density of caspase3^+^/GFP^+^ hippocampal neurons under hypoxia or normoxia was evaluated. * *p*＜0.05, ** *p*＜0.01, *** *p*＜0.001.


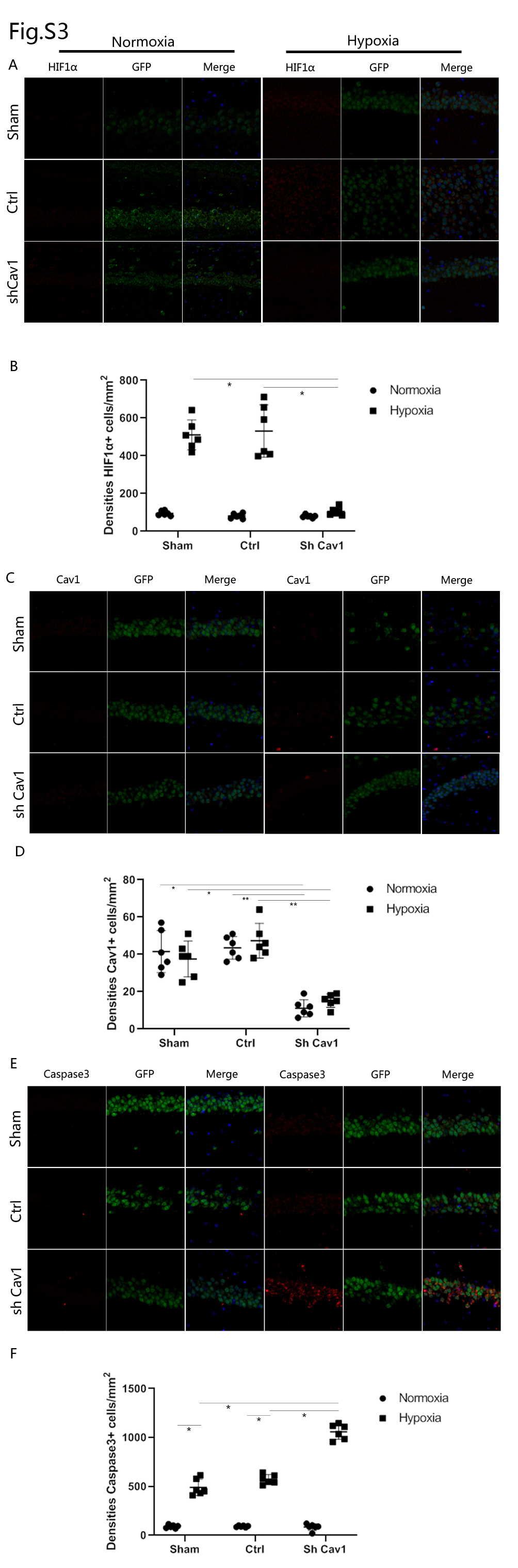


FIGURE S3 Roles of Cav-1 in regulating the apoptosis of Mice hippocampal neurons in Cav-1 downregulation mice hypoxia models. (A) Mice-transfected with sh-Cav-1 or LV-scramble RNA (ctrl) were kept under hypoxia or normoxia for 8 h, and Cav-1 levels in hippocampal neurons were then examined by immunohistochemistry. (B) The density of Cav-1^+^ hippocampal neurons under hypoxia or normoxia was evaluated. (C) Mice-transfected with sh-Cav-1 or LV-scramble RNA (ctrl) were kept under hypoxia or normoxia for 8 h, and HIF-1α levels in hippocampal neurons were then examined by immunohistochemistry. (D) The density of HIF-1α^+^ hippocampal neurons under hypoxia or normoxia was evaluated. (Scale bars, 10 μm) The results are presented as the mean ± SEM (n=6 animals per group). (E) Mice-transfected with sh-Cav-1 or LV-scramble RNA (ctrl) were kept under hypoxia or normoxia for 8 h, and caspase-3 levels in hippocampal neurons were then examined by immunohistochemistry. (F) The density of caspase3^+^ hippocampal neurons under hypoxia or normoxia was evaluated. * *p*＜0.05, ** *p*＜0.01.


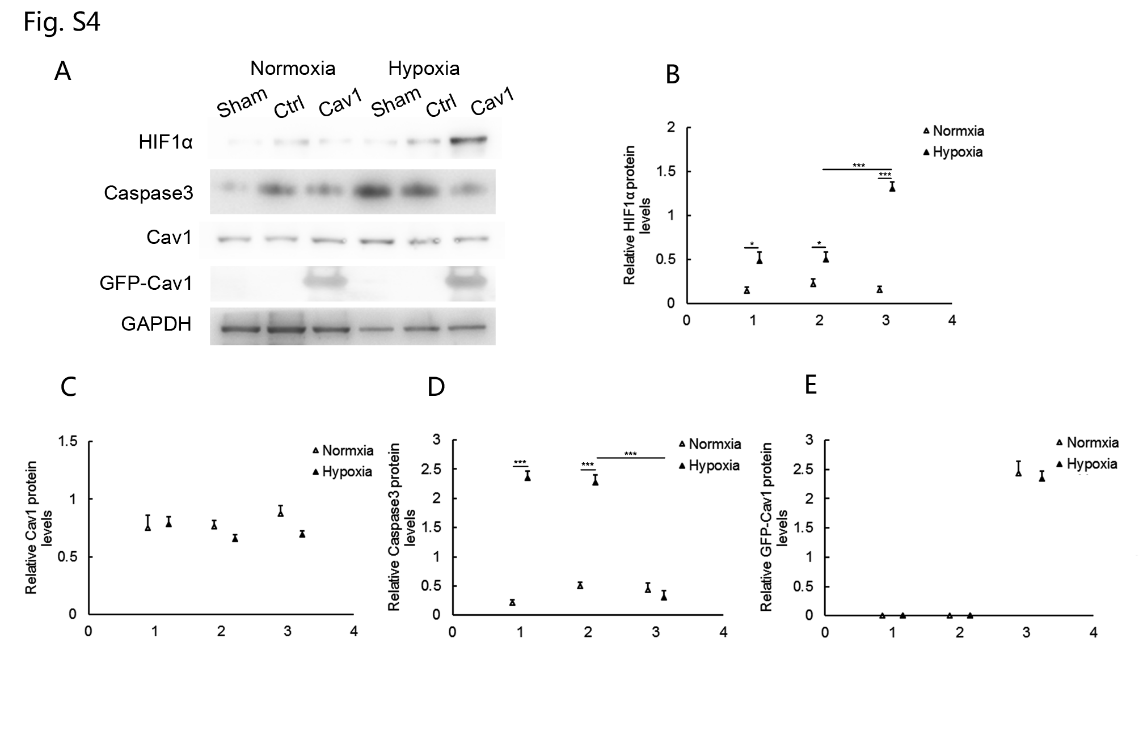


FIGURE S4 Roles of Cav-1 in regulating Mice hippocampal neuron apoptosis levels analysed by western blotting in Mice hypoxia models. (A) Mice-transfected Cav-1 or LV-GFP (ctrl) were maintained under hypoxia or normoxia for 8 h, and hippocampal neurons were subsequently examined by immunohistochemistry using anti-HIF-1α, Cav-1, and caspase3 antibodies. (B-E) The relative protein levels of HIF1α, caspase3, Cav-1 and GFP under hypoxia or normoxia were evaluated. The results are presented as the mean ± SEM (n=6 animals per group). *** *p*＜0.001.


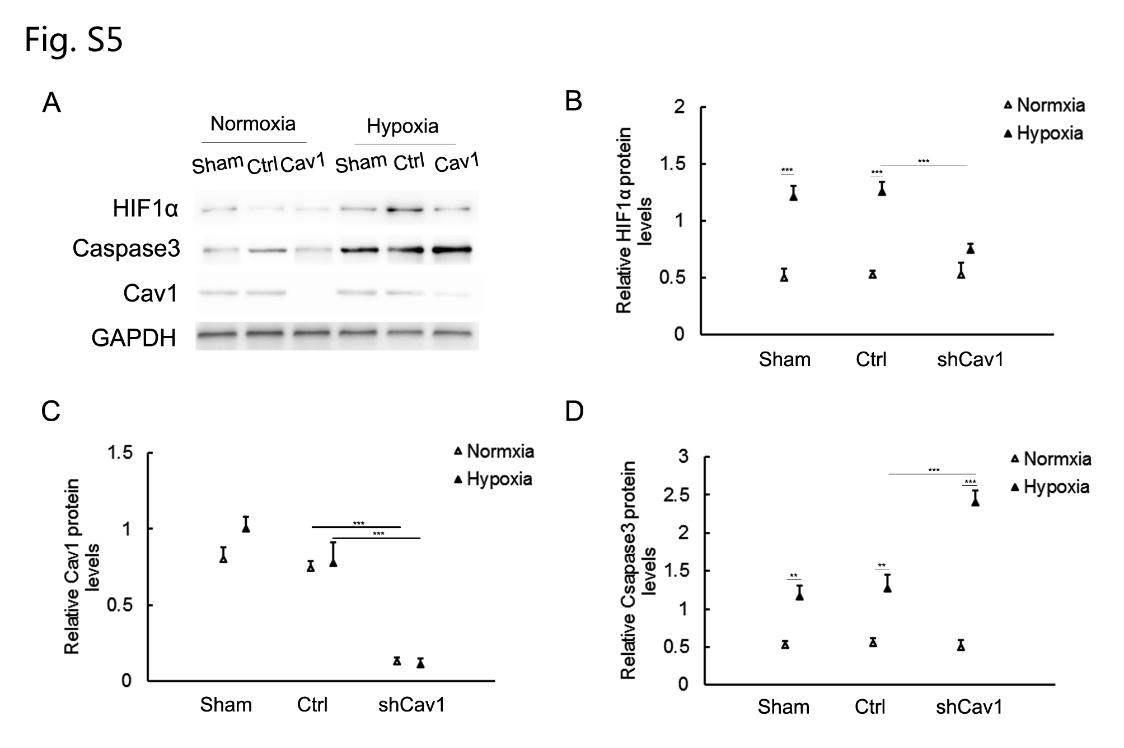


FIGURE S5 Roles of Cav-1 in regulating Mice hippocampal neuron apoptosis levels analysed by western blotting in NMR hypoxic models. (A) Mice-transfected ctrl or LV-sh-Cav1 cells were maintained under hypoxia or normoxia for 8 h, and hippocampal neurons were subsequently examined by immunohistochemistry using anti-caspase3, HIF-1α, and Cav-1 antibodies. (B-D) The relative protein levels of HIF1α, caspase3 and Cav-1 under hypoxia or normoxia were evaluated. The results are presented as the mean ± SEM (n=6 animals per group). ** *p*＜0.01, ****p*＜0.001.
